# Pretransplant Serum Hepatitis C Virus RNA Levels Predict Response to Antiviral Treatment after Living Donor Liver Transplantation

**DOI:** 10.1371/journal.pone.0058380

**Published:** 2013-03-07

**Authors:** Yoshihide Ueda, Toshimi Kaido, Yasuhiro Ogura, Kohei Ogawa, Atsushi Yoshizawa, Koichiro Hata, Yasuhiro Fujimoto, Aya Miyagawa-Hayashino, Hironori Haga, Hiroyuki Marusawa, Satoshi Teramukai, Shinji Uemoto, Tsutomu Chiba

**Affiliations:** 1 Department of Gastroenterology and Hepatology, Graduate School of Medicine, Kyoto University, Kyoto, Japan; 2 Department of Surgery, Graduate School of Medicine, Kyoto University, Kyoto, Japan; 3 Department of Diagnostic Pathology, Kyoto University Hospital, Kyoto, Japan; 4 Division of Clinical Trial Design and Management, Translational Research Center, Kyoto University Hospital, Kyoto, Japan; Kobe University, Japan

## Abstract

**Background:**

Given the limited efficacy and high adverse event rate associated with treatment of recurrent hepatitis C after liver transplantation, an individualized treatment strategy should be considered. The aim of this study was to identify predictors of response to antiviral therapy for hepatitis C after living donor liver transplantation (LDLT) and to study the associated adverse events.

**Methods:**

A retrospective chart review was performed on 125 hepatitis C virus (HCV)-positive LDLT recipients who received interferon plus ribavirin and/or peginterferon plus ribavirin therapy at Kyoto University between January 2001 and June 2011.

**Results:**

Serum HCV RNA reached undetectable levels within 48 weeks in 77 (62%) of 125 patients, and these patients were defined as showing virological response (VR). Of 117 patients, 50 (43%) achieved sustained VR (SVR). Predictive factors associated with both VR and SVR by univariate analysis included low pretransplant serum HCV RNA levels, a non-1 HCV genotype, and low pretreatment serum HCV RNA levels. In addition, LDLT from ABO-mismatched donors was significantly associated with VR, and white cell and neutrophil counts before interferon therapy were associated with SVR. Multivariate analysis showed that 2 variables–pretransplant serum HCV RNA level less than 500 kIU/mL and a non-1 HCV genotype–remained in models of both VR and SVR and that an ABO mismatch was associated with VR. No variables with a significant effect on treatment withdrawal were found.

**Conclusions:**

Virological response to antiviral therapy in patients with hepatitis C recurring after LDLT can be predicted prior to transplant, based on pretransplant serum HCV-RNA levels and HCV genotype. LDLT from ABO-mismatched donors may contribute to more efficacious interferon therapy.

**Trial Registration:**

UMIN-CTR UMIN000003286

## Introduction

Hepatitis C virus (HCV) infection, leading to liver cirrhosis and hepatocellular carcinoma, is the leading indications for liver transplantation in Japan, the United States, and Western Europe. However, almost all patients who undergo liver transplantation for HCV-related liver disease develop recurrent viral infection, and 70–90% of patients suffer from histologically proven recurrent hepatitis [1], [2], [3], [4], [5], [6]. The progression of recurrent hepatitis C is often accelerated and, without appropriate antiviral therapy, 10–25% of patients develop cirrhosis within 5 years after transplantation, resulting in poorer prognoses for HCV-positive recipients than HCV-negative recipients [7]. To prevent the progression of hepatitis C after liver transplantation, interferon-based combination therapy is commonly administered [8],[9]. However, its efficacy in liver transplant recipients is limited, with the mean sustained virological response (SVR) rate among patients with recurrent hepatitis C after liver transplantation being only 30% (range, 8–50%) [10]. One of the reasons for the low SVR rate is the high rate of treatment withdrawal. Several severe adverse events have been reported in transplant recipients after interferon therapy, including chronic rejection and *de novo* autoimmune hepatitis [11], [12], [13].

To improve the efficacy of anti-HCV treatment in patients with hepatitis C after liver transplantation, an individualized treatment strategy based on efficacy prediction and adverse events should be attempted. In several studies, an analysis of predictors associated with SVR was conducted in patients with recurrent hepatitis C after deceased donor liver transplantation (DDLT) [10], [14], . In these studies, variables most frequently associated with SVR were early virological response (EVR) at 3 months of therapy, HCV genotype 2, adherence to therapy, and baseline viremia [14], [15], [16], [17], [18], [19], [20]. Of these factors, EVR and adherence to therapy can only be recognized after the initiation of treatment. However, to enable decisions on treatment indications and strategy, predictors of response that are available before initiation of therapy are more valuable. Thus, an individualized treatment strategy could be based on the identification of baseline predictive factors before interferon therapy. Moreover, no study of factors predictive of response to the interferon therapy in patients with recurrent hepatitis C after living donor liver transplantation (LDLT) has been reported so far. Characteristics specific to LDLT, including blood-relative donors, post-transplant liver regeneration, and ABO-incompatible liver transplantation, might cause the antiviral effects of interferon therapy in these patients to differ from those who received DDLT.

The direct-acting antiviral agents telaprevir and boceprevir recently became available for clinical use. The results of clinical trials of these agents in combination with peginterferon plus ribavirin in nontransplant patients with HCV were promising [21], [22], [23], [24]. SVR rates to telaprevir-based combination therapy were significantly higher than those to the peginterferon-ribavirin combination. The efficacy in the patients who had suffered a relapse after a previous treatment by peginterferon plus ribavirin was especially striking [21], [24]. The SVR rate to telaprevir based-therapy in patients who had a previous relapse was more than 80%, while that in patients who had no response to previous treatment was around 30% [24]. These results suggest that patients who show a virological response (VR) to peginterferon plus ribavirin are expected to achieve SVR after telaprevir-based therapy. Therefore, identification of factors predictive of virological response to peginterferon plus ribavirin should also prove useful when making the clinical decision about telaprevir usage. In liver transplant recipients, the use of telaprevir and boceprevir poses risks because of their inhibitory action on the enzyme cytochrome P450 3A, responsible for the metabolism of both tacrolimus and cyclosporine. In fact, the phase I study of telaprevir in healthy individuals revealed that it significantly increased the blood concentrations of both tacrolimus and cyclosporine [25]. Therefore, the selection of the patients for whom telaprevir is prescribed is especially important in liver transplant recipients.

Recently, a polymorphism in the interleukin-28B (IL28B) gene region, encoding interferon-lambda 3, was identified as a strong predictive factor for response to antiviral treatment in nontransplant patients with hepatitis C [26], [27], [28]. In post-transplant patients, the IL28B polymorphism in both recipients and donors was shown to be associated with response to antiviral treatment [29], [30]. In addition, HCV-RNA mutations, including those affecting amino acid (aa) residues 70 and 91 in the core region of HCV and those in the interferon sensitivity determining region (ISDR) in nonstructural protein 5A (NS5A), were also demonstrated to be predictors of response to interferon therapy in transplant recipients, as well as in nontransplant settings [31], [32], [33]. These factors could be used to predict response to antiviral therapy, but these are presently not part of a routine clinical examination and require special techniques not covered by health insurance. Moreover, probing individual genetic information poses potential ethical issues.

The aims of this study were, therefore, to identify noninvasively obtained regular baseline factors associated with VR, SVR, and treatment withdrawal, in order to elucidate the factors associated purely with response to interferon therapy, to identify the valuables related to final outcomes, and to clarify the factors associated with adverse events.

## Methods

A retrospective chart review was performed for all HCV-positive liver transplant patients who received antiviral therapy with standard interferon and/or pegylated interferon in combination with ribavirin after liver transplantation at Kyoto University between January 2001 and June 2011.

### Patients

Between March 1999 and June 2011, 214 HCV-positive recipients underwent LDLT at Kyoto University. Of these, 157 patients were followed up for more than 6 months after LDLT in our hospital. Anti-viral therapy was administered to 125 of the 157 patients with recurrent hepatitis C between January 2001 and June 2011. The remaining 32 patients did not receive anti-viral therapy for various reasons: serum HCV-RNA negative after LDLT (n = 4), no histological hepatitis C recurrence in the follow-up period (n = 13), no fibrosis seen by liver histology (n = 8), and ongoing treatment for the other complications (n = 7). HCV RNA concentrations and histological evidence were used to diagnose patients with recurrent hepatitis C after LDLT. These patients were given combination therapies with interferon plus ribavirin and/or peginterferon plus ribavirin at Kyoto University between January 2001 and June 2011. The study protocol was approved by the Ethics Committee at Kyoto University and performed in compliance with the Helsinki Declaration. Written informed consent for participation in this study was not obtained, because this study is an observational study without use of human specimen. Our institutional review board waived the need for written informed consent from the participants of the initial study.

### Treatment Protocol and Definition of Responses to Treatment

Between January 2001 and April 2004, patients with recurrent hepatitis C after LDLT received treatment with interferon-α-2b (3 or 6 mega units, 3 times/week) plus ribavirin (400–800 mg/day orally), for the first 6 months. This was followed by interferon monotherapy for 6 months [34]. Forty patients received this treatment. Of the 40 patients, 14 patients achieved SVR and 9 withdrew from the treatment protocol. The remaining 17 patients, including 2 who relapsed and 15 nonresponders were retreated by the following protocol with peginterferon and rebavirin. Between May 2004 and June 2011, patients received combination therapy with peginterferon-α-2b (1.5 µg/kg) plus ribavirin (400–800 mg/day orally) [35]. Patients who acquired a negative serum HCV RNA status within 12 months after treatment initiation continued to receive the treatment for an additional 12 months before treatment termination. Total 102 patients, including 17 patients who had previously treated with standard interferon plus ribavirin and did not achieve SVR, were treated with this treatment protocol. Patients who were negative for serum HCV RNA for more than 6 months after completion of interferon therapy were defined as having achieved SVR. If serum HCV RNA was positive after 12 months of treatment, therapy was discontinued or switched to maintenance therapy with low-dose peginterferon [36], and the patient was classified as having shown no response. Treatment was discontinued in patients with severe adverse events. Additionally, peginterferon treatment was discontinued when neutrophil and platelet counts fell below 500/µL and 30000/µL, respectively, and ribavirin was discontinued when hemoglobin levels fell below 8 g/dL.

We studied the final outcomes of the treatment with peginterferon plus ribavirin (n = 102) and with standard interferon plus ribavirin (n = 23).

### Histological Assessment

Liver biopsies were performed when patients’ alanine aminotransferase (ALT) levels were more than twice the normal upper limit, or at yearly intervals, with informed consent. Biopsy specimens were evaluated by 2 pathologists (H.H. and A.M-H.) with extensive experience in the pathology of liver transplantation. Necroinflammatory activity (A0–A3) and fibrosis stage (F0–F4) were assessed using METAVIR scores [37], [38]. Activity was graded as A0 (no activity), A1 (mild activity), A2 (moderate activity), or A3 (severe activity); Fibrosis was staged as F0 (no fibrosis), F1 (mild fibrosis), F2 (moderate fibrosis), F3 (severe fibrosis), or F4 (cirrhosis).

### Immunosuppression

Tacrolimus and low-dose steroid therapy were administered to induce immunosuppression in most patients [34]. Four patients received cyclosporine microemulsions instead of tacrolimus. Mycophenolate mofetil was administered to patients who experienced refractory rejection or required reduction of tacrolimus or cyclosporine doses due to adverse events. Patients who received ABO blood-type incompatible transplants were treated with rituximab, plasma exchange, and hepatic artery or portal vein infusion with prostaglandin E1 and methylprednisolone [39].

### Virological Assays

HCV genotype was determined using a genotyping system based on polymerase chain reaction (PCR) to amplify the core region using genotype-specific PCR primers [40]. Serum HCV RNA load was evaluated before LDLT, before interferon treatment, once a month during treatment, and 24 weeks after treatment, using PCR and an Amplicor HCV assay (Cobas Amplicor HCV Monitor, Roche Molecular Systems, Pleasanton, CA, USA) until April 2008, or a real-time PCR-based quantitation method for HCV (COBAS AmpliPrep/COBAS TaqMan HCV Test, Roche Molecular Systems, Pleasanton, CA, USA) from May 2008. Detection of amino acid substitutions in the HCV core region was performed using the method reported previously [31].

### Statistical Analysis

To evaluate the association between the patient characteristics and the outcomes (VR, SVR, or withdrawal), the Wald test was performed based on a logistic regression model. Multivariate logistic regression analysis with backward variable selection was used to identify independent and significant predictors for the outcomes, and to estimate the odds ratio (OR) ant its 95% confidence interval (CI). A p-value of 0.05 was used for variable selection and was regarded as significant. Statistical analyses were performed using SAS version 9.2 (SAS Institute Inc., Cary NC).

## Results

### Patient Characteristics

This study included 125 HCV-infected liver transplant patients treated with standard interferon and/or pegylated interferon in combination with ribavirin for recurrent hepatitis C after LDLT. Of the 125 patients, 69 (55%) were male, and the median age was 57 years (range: 15–70) at the beginning of the therapy. Most patients were infected with HCV genotype 1b (n = 103, 82%). HCV genotypes of the remaining patients were 2a (n = 13), 2b (n = 5), 3a plus 3b (n = 1), not determined (n = 2), and not examined (n = 1). Median serum HCV RNA load was 410 kIU/mL (range: <0.5–5000<kIU/mL) before LDLT, and 3260 kIU/mL (range: 31–69000<kIU/mL) at the beginning of the interferon therapy after LDLT. The median donor age was 41 (range: 19–65) years. Seventy-two donors (58%) were male, and 86 (69%) were related to the recipients. The graft type was the right lobe in 109 patients (87%), and the left lobe in 16 patients (13%). The blood type combination was incompatible in 26 patients (21%). The median time to treatment initiation after LDLT was 9.0 months (1.1–85.3 months). Before treatment, the necroinflammatory activity was A1 or greater in all patients, and 104 patients (83%) had a fibrosis score of F1 or greater (METAVIR score). Tacrolimus-based immunosuppression was used in 116 patients (93%). Among patients receiving tacrolimus for immunosuppression, the mean serum trough level was 6.0 ng/mL (range: 2.0–12.7) at the initiation of interferon therapy. In addition to calcineurin inhibitors, mycophenolate mofetil and prednisolone were used at the initiation of the interferon treatment in 36 (29%) and 19 (15%) patients, respectively.

### Efficacy of Interferon Therapy

**Figure 1:**
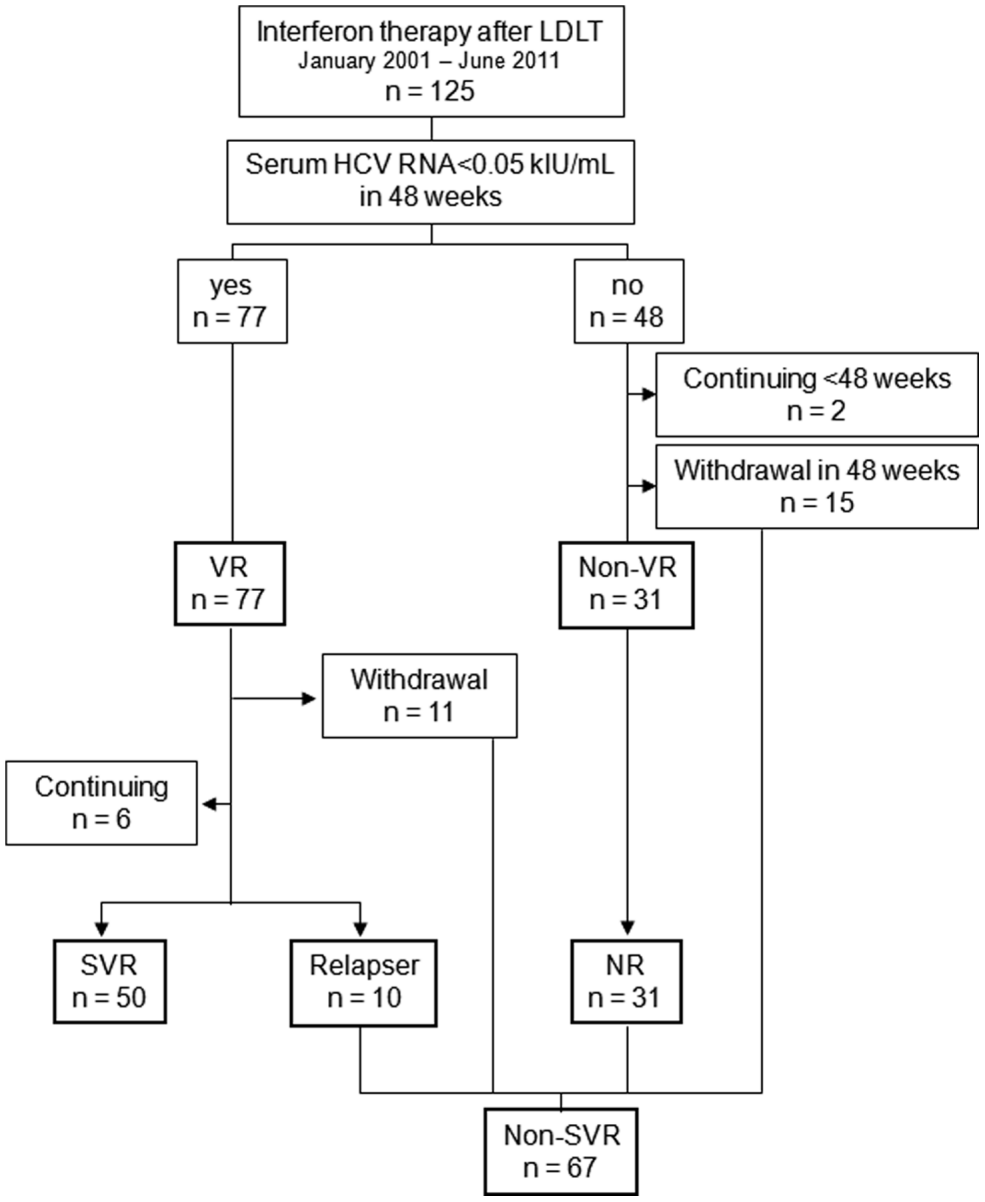
Flow diagram showing the outcome of interferon therapy in patients with recurrent hepatitis C after living donor liver transplantation (LDLT) and indicating the classification of patients in this study. N, number of patients; VR, virological response; SVR, sustained virological response; NR, nonresponder.

Of the 125 patients who received interferon therapy, serum HCV RNA reached undetectable levels (less than 0.05 kIU/mL) within 48 weeks in 77 patients (62%) (Figure 1). These patients were defined as showing virological response (VR). Of the remaining 48 patients, 2 patients received treatment for less than 48 weeks, and 15 patients withdrew from the treatment protocol within 48 weeks because of worsening of liver function (n = 5), recurrent hepatocellular carcinoma (n = 2), ascites (n = 2), anemia (n = 1), leucopenia (n = 1), brain hemorrhage (n = 1), biliary complication (n = 1), sepsis (n = 1), or myocardial infarction (n = 1). The remaining 31 patients with detectable HCV RNA in the serum 48 weeks after the initiation of the treatment were placed in the non-VR group. All patients in the non-VR group received peginterferon plus ribavirin therapy, including 9 patients who had previously treated with standard interferon plus ribavirin and did not achieve SVR. Of the patients with VR, 11 discontinued the treatment protocol within 24 weeks after serum HCV-RNA became negative, and 6 patients are still under treatment. The reasons for discontinuation were biliary complications (n = 2), worsening of liver function (n = 2), general fatigue (n = 2), recurrent hepatocellular carcinoma (n = 1), leucopenia (n = 1), hemoptysis (n = 1), brain tumor (n = 1), and depression (n = 1). Of 60 patients who achieved VR and completed the treatment protocol, 50 achieved SVR and 10 relapsed. None of the non-VR patients achieved VR even after more than 48 weeks of treatment, and were classified as nonresponder (NR).

In summary, among the 117 patients in whom the final outcomes of the treatment could be evaluated, 50 patients (43%) achieved SVR, and the remaining 67 patients, including 10 who relapsed (9%), 31 NR (26%), and 26 withdrawals (22%), were classified as non-SVR.

### Factors Predictive of Virological Response

Factors that could predict virological response were analyzed by comparing patients in the VR (n = 77) and non-VR (n = 31) groups (Table 1). Univariate analysis demonstrated that a low pretransplant serum HCV RNA level (less than 500 kIU/mL, *P*<0.001; and less than 1000 kIU/mL, *P*<0.001), an ABO-mismatched donor (*P* = 0.036), HCV genotype (non-1, *P* = 0.001), and a low pretreatment serum HCV RNA level (less than 5000 kIU/mL, *P* = 0.020) were significantly associated with VR. There were no significant associations with any other variables, including donor factors. Multivariate analysis revealed that the 3 variables that retained a significant association in the model were a pretransplant serum HCV RNA level less than 500 kIU/mL [odds ratio (OR): 0.178, 95% confidence interval (CI): 0.054–0.535, *P* = 0.001], a non-1 HCV genotype (OR: 0.087, 95% CI: 0.000–0.589, *P* = 0.008), and an ABO-mismatched donor (OR: 5.492, 95% CI: 1.004–58.06, *P* = 0.049) (Table 2). All 20 patients with a non-1 HCV genotype achieved VR, while VR rate in patients with the HCV genotype 1 was 65% (57 out of 88 patients). In the patients with HCV genotype 1, VR rate was 80% (36 of 45 patients) when pretransplant serum HCV-RNA level was less than 500 kIU/mL and 42% (15 of 36 patients) when it was 500 kIU/mL or more. Among 22 recipients from ABO-mismatched donors, 20 patients (91%) showed VR, while 57 (66%) out of 86 patients who underwent LDLT from an ABO-matched (identical and compatible) donor achieved VR.

**Table 1:** Baseline predictive factors before liver transplantation (pre-LT), at liver transplantation (at LT), and before interferon therapy (pre-IFN) associated with virorogical response (VR) and sustained VR (SVR): Univariate analysis.

		VR	non-VR	*P*	SVR	non-SVR	*P*
		n = 77	n = 31		n = 50	n = 67	
Age at LT (years)		55 (8−67)	56 (37−69)	0.462	54.5 (8−67)	56 (30−69)	0.212
Gender	Male	45 (74%)	16 (26%)	0.518	30 (46%)	35 (54%)	0.404
	Female	32 (68%)	15 (32%)		20 (38%)	32 (62%)	
HCC pre-LT	No	29 (71%)	12 (29%)	0.919	18 (43%)	24 (57%)	0.984
	Yes	48 (72%)	19 (28%)		32 (43%)	43 (57%)	
MELD pre-LT		15.5 (3−51)	15 (6−25)	0.403	16 (3−51)	15 (0−43)	0.616
Child-Pugh pre-LT	A/B	35 (74%)	12 (26%)	0.488	25 (49%)	26 (51%)	0.192
	C	41 (68%)	19 (32%)		24 (37%)	41 (63%)	
	unknown	1	0		1	0	
Serum HCV RNA pre-LT	<100 kIU/mL	16 (89%)	2 (11%)	0.063	11 (65%)	6 (35%)	0.028
	100 kIU/mL≤	52 (65%)	28 (35%)		31 (35%)	57 (65%)	
	unknown	9	1		8	4	
Serum HCV RNA pre-LT	<500 kIU/mL	50 (85%)	9 (15%)	<0.001	30 (55%)	25 (45%)	0.002
	500 kIU/mL≤	18 (46%)	21 (54%)		12 (24%)	38 (76%)	
	unknown	9	1		8	4	
Serum HCV RNA pre-LT	<1000 kIU/mL	56 (81%)	13 (19%)	<0.001	34 (49%)	36 (51%)	0.013
	1000 kIU/mL≤	12 (41%)	17 (59%)		8 (23%)	27 (77%)	
	unknown	9	1		8	4	
HCV genotype	Non-1	20 (100%)	0 (0%)	0.001	15 (79%)	4 (21%)	0.002
	1	57 (65%)	31 (35%)		35 (36%)	62 (64%)	
	unknown				0	1	
Donor age at LT (years)		42 (20−63)	38 (21−61)	0.504	43 (20−60)	38 (19−63)	0.748
Donor gender at LT	Male	41 (67%)	20 (33%)	0.287	27 (40%)	40 (60%)	0.538
	Female	36 (77%)	11 (23%)		23 (46%)	27 (54%)	
Sex mismatch	Match	28 (72%)	11 (28%)	0.932	18 (43%)	24 (57%)	0.984
	Mismatch	49 (71%)	20 (29%)		32 (43%)	43 (57%)	
ABO mismatch	Match	57 (66%)	29 (34%)	0.036	38 (40%)	56 (60%)	0.310
	Mismatch	20 (91%)	2 (9%)		12 (52%)	11 (48%)	
Relation of donor	Nonrelated	24 (73%)	9 (27%)	0.827	16 (44%)	20 (56%)	0.803
	Related	53 (71%)	22 (29%)		34 (42%)	47 (58%)	
Graft type	Left lobe	13 (81%)	3 (19%)	0.347	8 (62%)	5 (38%)	0.155
	Right lobe	64 (70%)	28 (30%)		42 (40%)	62 (60%)	
Splenectomy	No	38 (68%)	18 (32%)	0.413	25 (39%)	39 (61%)	0.378
	Yes	39 (75%)	13 (25%)		25 (47%)	28 (53%)	
Age pre-IFN (years)		57 (15−68)	57 (41−70)	0.494	56 (15−68)	57 (32−70)	0.200
Months from LT to therapy		9.2 (1.1−85.3)	8.9 (1.8−59.0)	0.846	9.0 (1.3−85.3)	9.0 (1.3−72.4)	0.879
Trough level for tacrolimus (ng/mL)pre-IFN		5.9 (2.0−10.9)	6.4 (3.3−10.6)	0.323	6.2 (2.2−9.5)	5.9 (2.0−12.7)	0.933
MMF pre-IFN	No	55 (71%)	23 (29%)	0.772	36 (43%)	48 (57%)	0.966
	Yes	22 (73%)	8 (27%)		14 (42%)	19 (58%)	
Prednisolone pre-IFN	No	64 (70%)	28 (30%)	0.347	41 (41%)	60 (59%)	0.245
	Yes	13 (81%)	3 (19%)		9 (56%)	7 (44%)	
Serum HCV RNA pre-IFN	<1000 kIU/mL	17 (89%)	2 (11%)	0.064	8 (38%)	13 (62%)	0.583
	1000 kIU/mL≤	58 (67%)	29 (33%)		42 (45%)	52 (55%)	
	unknown	2	0		0	2	
Serum HCV RNA pre-IFN	<5000 kIU/mL	52 (78%)	15 (22%)	0.020	36 (50%)	36 (50%)	0.030
	5000 kIU/mL≤	18 (55%)	15 (45%)		10 (28%)	26 (72%)	
	unknown	7	1		4	5	
White cell count (102/mL)		51 (13−114)	49 (17−98)	0.135	49 (18−114)	48.5 (13−99)	0.049
Neutrophil count (102/mL)		26 (8−89)	22 (11−58)	0.127	26 (11−89)	23 (8−61)	0.044
Hemoglobin (g/dL)		12.0 (9.2−17.2)	12.0 (8.9−17.9)	0.638	12.0 (9.4−17.2)	11.8 (8.9−17.9)	0.157
Platelet count (104/mL)		21.7 (4.7−58.1)	15.1 (4.3−40.0)	0.153	20.3 (5.0−58.1)	15.8 (4.3−45.8)	0.165
AST (IU/L)		78 (19−352)	72 (25−464)	0.677	85 (21−352)	75 (24−547)	0.887
ALT (IU/L)		93 (18−395)	82 (21−392)	0.544	106 (22−395)	82 (18−597)	0.251
ALP (IU/L)		461 (199−1985)	433 (168−2977)	0.345	470 (204−1985)	470 (168−2977)	0.610
g-GTP (IU/L)		118.5 (15−1623)	114 (20−1827)	0.856	141 (15−1623)	115 (20−1827)	0.356
Bilirubin (mg/dL)		0.9 (0.3−11.0)	0.9 (0.3−10.4)	0.827	0.9 (0.4−11.0)	1.0 (0.3−13.7)	0.611
Activity grade pre-IFN	A1	54 (75%)	18 (25%)	0.448	35 (47%)	40 (53%)	0.517
	A2	22 (65%)	12 (35%)		14 (36%)	25 (64%)	
	A3	1 (50%)	1 (50%)		1 (33%)	2 (67%)	
Fibrosis stage pre-IFN	F0	9 (60%)	6 (40%)	0.446	6 (32%)	13 (68%)	0.530
	F1	54 (75%)	18 (25%)		34 (46%)	40 (54%)	
	F2/3	14 (67%)	7 (33%)		10 (42%)	14 (58%)	
Steatosis (5%<) pre-IFN	No	40 (69%)	18 (31%)	0.609	27 (42%)	38 (58%)	0.633
	Yes	36 (73%)	13 (27%)		23 (46%)	27 (54%)	
	unknown	1	0		0	2	
Cholestasis pre-IFN	No	58 (71%)	24 (29%)	0.903	38 (42%)	53 (58%)	0.577
	Yes	18 (72%)	7 (28%)		12 (48%)	13 (52%)	
	unknown	1	0		0	1	

NOTE. Qualitative variables are shown in number; and quantitative variables expressed as median (range). P-values are calculated by Wald test for logistic regression analysis.

LT, liver transplantation; HCC, hepatocellular carcinoma; MELD, model for end-stage liver disease; HCV, hepatitis C virus; MMF, mycophenolate mofetil; AST, aspartate aminotransferase; ALT, alanine aminotransferase; ALP, alkaline phosphatase; g-GTP, gamma-glutamyl transpeptidase.

**Table 2:** Predictive factors associated with virological response (VR): Multivariate analysis.

		Odds Ratio	95% confidence intervals	P-value
Serum HCV RNA pre-LT	<500 kIU/mL	1	-	-
	500 kIU/mL≤	0.178	0.054–0.535	0.001
HCV genotype	Non-1	1	-	-
	1	0.087	0.000–0.589	0.008
ABO mismatch	Match	1	-	-
	Mismatch	5.492	1.004-58.06	0.049

HCV, hepatitis C virus; LT, liver transplantation.

### Factors Predictive of SVR

The same variables were analyzed to clarify factors that predicted SVR by comparing patients in the SVR (n = 50) and non-SVR (n = 67) groups (Table 1). By univariate analysis, the same variables that had a significant association with VR were identified as significant predictive factors for SVR–low pretransplant serum HCV RNA levels (less than 100 kIU/mL, *P* = 0.028; less than 500 kIU/mL, *P* = 0.002; and less than 1000 kIU/mL, *P* = 0.013), HCV genotype (non-1, *P* = 0.002), and low pretreatment serum HCV RNA levels (less than 5000 kIU/mL, *P* = 0.030). In addition, white cell count (*P* = 0.049) and neutrophil count (*P* = 0.044) before interferon therapy were significantly associated with SVR. Multivariate analysis showed that 2 variables were independently associated with SVR–a non-1 HCV genotype (OR: 0.182, 95% CI: 0.054–0.614, *P* = 0.006), and pretransplant serum HCV RNA levels lower than 500 kIU/mL (OR: 0.310, 95% CI: 0.130–0.742, *P* = 0.009) (Table 3). SVR rate among patients with a non-1 HCV genotype was 79% (15 of 19 patients) on average, 83% (10 of 12 patients) when pretransplant serum HCV-RNA level was less than 500 kIU/mL, and 50% (2 of 4 patients) when it was 500 kIU/mL or more. In patients with HCV genotype 1, SVR rate was 36% (35 of 97 patients) on average, 47% (20 of 43 patients) when pretransplant serum HCV-RNA level was less than 500 kIU/mL, and 22% (10 of 45 patients) when it was 500 kIU/mL or more.

**Table 3:** Predictive factors associated with sustained virological response (SVR): Multivariate analysis.

		Odds Ratio	95% confidence intervals	P-value
HCV genotype	Non-1	1	-	-
	1	0.182	0.054–0.614	0.006
Serum HCV RNA pre-LT	<500 kIU/mL	1	-	-
	500 kIU/mL≤	0.310	0.130–0.742	0.009

HCV, hepatitis C virus; LT, liver transplantation.

### Amino Acid Substitutions in Core Region of HCV

To determine the viral factors that predicted VR and SVR in patients infected with HCV genotype 1b, association of aa substitutions at aa 70 of arginine or glutamine/histidine and aa 91 of leucine or methionine with VR and SVR were analyzed in 40 patients, whose pre-treatment sera were stored (Table 4). As a result, substitutions of both aa 70 and aa 91 were not significantly associated with VR and SVR.

**Table 4:** Association of amino acid substitutions in the core region with virorogical response (VR) and sustained VR (SVR) in 40 patients infected with HCV genotype 1b: Univariate analysis.

		VR	non-VR	*P*	SVR	non-SVR	*P*
		n = 22	n = 13		n = 14	n = 24	
Core aa 70	Arg	9 (75%)	3 (25%)	0.289	7 (50%)	7 (50%)	0.204
	Gln/His	13 (57%)	10 (43%)		7 (29%)	17 (71%)	
Core aa 91	Leu	14 (64%)	8 (36%)	0.902	9 (38%)	15 (63%)	0.912
	Met	8 (62%)	5 (38%)		5 (36%)	9 (64%)	
Core aa 70 and 91	70 Arg and 91 Leu	6 (67%)	3 (33%)	0.784	5 (50%)	5 (50%)	0.320
	Others	16 (62%)	10 (38%)		9 (32%)	19 (68%)	
Core aa 70 and 91	70 Gln/His and 91 Met	5 (50%)	5 (50%)	0.324	3 (30%)	7 (70%)	0.603
	Others	17 (68%)	8 (32%)		11 (39%)	17 (61%)	

NOTE. Data are shown in number. P-values are calculated by Wald test for logistic regression analysis.

Arg, Arginine; Gln, glutamine; His, histidine; Leu, leucine; Met, methionine.

### Predictors of Withdrawal from Therapy

Predictive factors for withdrawal from the treatment protocol were evaluated by comparing 26 patients who withdrew from the treatment protocol and the patients who completed the treatment including patients with SVR, patients who relapsed, and NR. None of the variables analyzed had a significant effect on withdrawal (Data not shown).

## Discussion

In this study, we identified 2 independent predictors of SVR in patients with recurrent hepatitis C after LDLT by multivariate analysis: A non-1 HCV genotype and pretransplant serum HCV-RNA levels lower than 500 kIU/mL. The same factors were identified as predictors for VR, which purely indicates response to interferon therapy, by excluding the influences of the premature termination of the therapy and virological relapse after termination of the treatment. In addition, an ABO-incompatible LDLT was identified as an independent variable predicting VR.

In non-transplant settings, pretreatment predictors of response to interferon therapy have been analyzed in many studies, and the viral genotype and pretreatment viral load have been almost invariably shown to be 2 major predictors of SVR [41], [42], [43], [44]. SVR rates were higher in patients infected with a non-1 HCV genotype and in those with a low pretreatment viral load. These 2 factors have been also identified in several reports [16], [17], [18], [19] as factors predicting SVR in patients with recurrent hepatitis C after DDLT. In the present study, a non-1 HCV genotype was again identified as an independent predictive factor for both VR and SVR in patients with recurrent hepatitis C after LDLT by multivariate analysis. A pretreatment viral load <5000 kIU/mL was also a significant predictive factor by univariate analysis, but it was not an independently associated variable by multivariate analysis. On the other hand, pretransplant viral load was identified as an independent variable predictive of both VR and SVR by multivariate analysis.

While reports of factors that can control viral load exist, the mechanism by which serum HCV-RNA levels are regulated has not yet been completely clarified. A correlation between mutations in the ISDR sequence in the NS5A region of the HCV genome and serum HCV RNA levels has been reported. We did not analyze this viral factor in the current study; however, it is possible that the HCV genome sequence determines both pretransplant viremia and response to interferon therapy. The host polymorphism in IL28B, which was identified as a strong predictor of virological response to interferon therapy in patients with hepatitis C, was recently reported to be associated with baseline viral load [26], [45]. The allele associated with a better treatment response is associated with a higher baseline viral load. This finding does not correspond with our results showing that a low HCV load predicts a better response to treatment. We speculate that the balance between host immunity and HCV replication regulates the serum HCV load, and that this balance also determines VR. As pretreatment viral load in post-transplant patients is influenced by immunosuppressive agents, the original host-virus balance would be reflected better by serum HCV levels before transplantation than by those after transplantation. It is unclear whether this result is specific to LDLT or holds true for both DDLT and LDLT. The significance of pretransplant viral load in DDLT as a predictor for virological response to post-transplant interferon therapy has not been analyzed in most previous studies [10]. Further analysis in patients who receive DDLT could help clarify the underlying mechanism.

Liver transplantation across the ABO blood-type barrier (ABO-incompatible) is generally contraindicated because of the possibility of graft loss caused by antibody-mediated rejection and is performed under exceptional circumstances as a rescue option in an emergent situation. However, ABO-incompatible LDLT has been performed in Japan to overcome organ shortage problems. Recently, rituximab prophylaxis and local infusion of prostaglandin E1 and steroids were established as therapeutic measures for recipients who underwent ABO-incompatible LDLT, and these treatments improved outcomes [46]. Interestingly, in this study, we found that an ABO-mismatched donor is associated with VR to interferon therapy. The reason for this interesting finding is unclear, but it is possible that either subclinical antibody-mediated rejection or drugs such as rituximab and prostaglandin E1 used in ABO-incompatible recipients may contribute to the higher VR to interferon therapy. There is hope that future studies to clarify the basic mechanism underlying this result will lead to a novel strategy to improve the efficacy of interferon therapy in patients with hepatitis C.

Amino acid substitutions of core region of HCV were not associated with treatment response in our analysis. We do not know the reason for the difference of impact of substitution of core aa 70 and aa 91 on virological response to interferon therapy from a previous report, in which SVR rate were significantly higher in transplant recipients with aa 70 of arginine and aa 91 of leucine of core region of HCV [33]. As sample size of both the previous study and our present study are small, and our present study did not assess the other HCV RNA mutations, including ISDR [32] and interferon/ribavirin resistance-determining region [47] in NS5A, and IL28B polymorphism in recipients and donors, further analysis should be required in larger cohorts.

Another aim of this study was to identify predictive variables for adverse events during interferon therapy, but none of the studied factors proved to be statistically significant predictors of withdrawal from the treatment protocol. As patients withdrew from the treatment for diverse reasons, it would be difficult to predict each adverse event before the initiation of interferon therapy. Therefore, careful follow-up during the treatment procedure is important for early detection of adverse events and to prevent progression to severe complications.

In this study, the final outcomes of the treatment including standard interferon plus ribavirin and peginterferon plus ribavirin were analyzed. Difference of the efficacy between standard interferon and peginterferon might affect the results of our present study. We predicted that patients who had virological response to standard interferon would also show the same response to peginterferon, because it is reported that the efficacy of peginterferon plus ribavirin is higher than that of standard interferon plus ribavirin [44], [48]. Accordingly, the patients who achieved SVR by standard interferon were included in the present study. On the other hand, all nonresponders and all patients who relapsed by standard interferon plus ribavirin were retreated with peginterferon plus ribavirin, and we analyzed the final outcomes of the peginterferon plus ribavirin therapy. Therefore, we conclude that the difference of treatment regimen has little influence on our results.

In conclusion, SVR to antiviral therapy in patients with recurrent hepatitis C after LDLT is predictable before transplant by serum HCV-RNA level and HCV genotype. In addition, patients who undergo ABO-incompatible LDLT appear to have a better VR to interferon therapy after liver transplantation. Mechanisms underlying these interesting results are unknown at present, but these findings are likely to be useful for improved clinical assessment of patients with hepatitis C after liver transplantation, and could lead to development of new strategies for better outcomes in LDLT recipients with the HCV genotype 1 and/or a higher pretransplant viral load.
