# Correction: Pretransplant Serum Hepatitis C Virus RNA Levels Predict Response to Antiviral Treatment after Living Donor Liver Transplantation

**DOI:** 10.1371/annotation/1601274b-def0-4970-8e61-62e657c2d1ae

**Published:** 2013-11-13

**Authors:** Yoshihide Ueda, Toshimi Kaido, Yasuhiro Ogura, Kohei Ogawa, Atsushi Yoshizawa, Koichiro Hata, Yasuhiro Fujimoto, Aya Miyagawa-Hayashino, Hironori Haga, Hiroyuki Marusawa, Satoshi Teramukai, Shinji Uemoto, Tsutomu Chiba

In Table 1, the counts for White cell count, Neutrophil count, and Platelet count as well as the unit of measurement are incorrect. The correct counts and measurements are White cell count (10^2 μL), Neutrophil count (10^2 μL), and Platelet count (10^4 μL). 

